# Evaluation of Epithelial Integrity in Human Precision‐Cut Kidney Slices

**DOI:** 10.1111/apm.70205

**Published:** 2026-04-10

**Authors:** Gitte A. Pedersen, Benjamin B. Green, Camilla Merrild, Søren H. Elsborg, Mia G. Madsen, Anna K. Keller, Henricus A. M. Mutsaers, Rikke Nørregaard, Lene N. Nejsum

**Affiliations:** ^1^ Department of Clinical Medicine Aarhus University Aarhus Denmark; ^2^ Department of Urology Aarhus University Hospital Aarhus Denmark; ^3^ Department of Renal Medicine Aarhus University Hospital Aarhus Denmark

## Abstract

Renal epithelial cells are pivotal in renal regulation of body fluid and electrolyte balance. While rodent models are commonly used to study renal physiology, species‐specific differences limit their translational relevance. To bridge this gap, we used human precision‐cut kidney slices (PCKS), an ex vivo tissue‐based model that maintains native tissue architecture and cellular diversity. This study had two aims: (1) to determine whether PCKS preserve short‐term aquaporin‐2 (AQP2) trafficking in response to desmopressin (dDAVP), and (2) to assess whether cortical PCKS maintain epithelial integrity during 24–48 h incubation. In medullary PCKS, 30 min dDAVP stimulation resulted in accumulation of AQP2 at the apical membrane, indicating preserved signaling. In cortical PCKS, AQP2 already showed substantial apical localization under baseline conditions, and no additional redistribution was detected after dDAVP stimulation. In cortical PCKS, incubation for 24 h and 48 h led to mislocalization of AQP2, the Na/K‐ATPase and uromodulin, and to altered localization of the adhesion proteins E‐cadherin and N‐cadherin and the tight junction protein ZO‐1 with a shift towards apical localization, indicating loss of epithelial integrity. Thus, short‐term signaling was preserved at 30 min, whereas epithelial transport and adhesion proteins became mislocalized after 24–48 h in human PCKS.

## 1 Introduction

The kidneys are vital in maintaining body fluid and electrolyte balance through filtration, reabsorption, and secretion processes. The human kidneys filter approximately 180 L of fluid per day, with plasma components passing through the glomerular barrier. The urinary filtrate undergoes processing via epithelial transport mechanisms that regulate ions, solutes, and water movement in the nephrons and collecting ducts to produce the final urine. These epithelial transport processes are tightly managed by epithelial transport proteins in the plasma membrane that control selective permeability and maintain tissue integrity. To study renal physiology and pathophysiology, in vivo rodent models are commonly used. However, while informative, rodent models do not fully recapitulate human kidney function due to species‐specific differences. Pig kidneys are closer to human kidneys, but their use is limited by cost, ethical considerations, and restricted experimental flexibility.

Less than 10% of drugs entering clinical trials fail to reach approval [1]. This discrepancy between results from the laboratory and the efficiency in clinical trials may be due to the lack of a translational model that translates observations made in experimental animals to patients. To address this limitation, we established the model of human precision‐cut kidney slices (PCKS), in which cellular diversity and organ architecture are maintained. PCKS are commonly used to study renal inflammation and fibrosis as well as a pre‐clinical drug screening platform [2, 3, 4, 5, 6]. However, it remains to be evaluated to what extent PCKS can be used to study renal epithelial physiology. Many cellular processes, such as regulated exocytosis, occur rapidly in response to external stimuli. One such example from the kidneys is aquaporin‐2 (AQP2) in collecting duct principal cells. AQP2 is localized in subapical transport vesicles and upon stimulation with the antidiuretic hormone vasopressin, AQP2 accumulates in the apical plasma membrane, instantly increasing collecting duct water permeability [7, 8]. This shuttling of AQP2 to the apical plasma membrane is pivotal in the ability of the kidneys to fine‐tune urine concentration. AQP2 trafficking has been studied in in vitro cell culture models as well as in vivo, especially in rodent models [9, 10, 11, 12, 13, 14, 15], complemented with ex vivo isolated collecting ducts [7] and slices of rodent kidneys, which would correspond to the human PCKS [13, 16]. Therefore, it is expected that processes such as AQP2 trafficking can also be studied in the human PCKS. Other processes, such as regulation of gene expression and modulation of tight junctions to regulate permeability, are slower and occur over hours to days, and it is so far unknown if the PCKS maintain epithelial integrity for that time period.

To evaluate which epithelial processes can be studied in human PCKS, we examined (i) short‐term dDAVP‐responsive AQP2 trafficking and (ii) maintenance of epithelial integrity during 24–48 h culture. Short‐term signaling was assessed by AQP2 redistribution after 30 min dDAVP stimulation, whereas epithelial integrity was evaluated at 24 and 48 h by subcellular localization of selected transport and junctional proteins using multiplex immunofluorescence [17].

## 2 Materials and Methods

### 2.1 Ethics Statement

The use of human kidney tissue to generate PCKS was approved by the Central Denmark Region Committees for Biomedical Research Ethics (Journal No. 1–10–72‐211‐17) and the Danish Data Protection Agency. All patients provided written informed consent.

### 2.2 Human Precision‐Cut Kidney Slices

The protocol for generating human PCKS has previously been described [18]. The tissue was sampled from tumor nephrectomies, and PCKS were generated using the Alabama R&D Tissue Slicer as previously described [6, 17, 18]. Tissue from 10 patients with functional (eGFR > 60 mL/min/1.73m^2^) and macroscopically healthy renal cortical or medullary tissue was included. After collection, the tissue was immediately placed in ice‐cold UW preservation buffer to limit warm ischemia time, and the time from tissue retrieval to slicing did not exceed 20 min.

The slicing procedure was performed in ice‐cold, carbogen‐saturated Krebs–Henseleit buffer supplemented with 25 mM NaHCO_3_, 25 mM D‐glucose, and 10 mM 4‐(2‐hydroxyethyl) piperazine‐1‐ethanesulfonic acid. Subsequently, the PCKS were cultured in William's E medium with GlutaMAX supplemented with 2.7 g/L D‐(+)‐glucose and 10 μg/mL ciprofloxacin at 37°C in an 80% O_2_ and 5% CO_2_ atmosphere with gentle shaking. Cortical slices were incubated for either 0 h, 24 h, or 48 h. For short‐term experiments, PCKS were incubated with desmopressin (dDAVP, 1 nM) for 30 min; controls were incubated with PBS/saline. The PCKS were subsequently fixed in 4% paraformaldehyde for 24 h at 4°C, dehydrated, and embedded in paraffin. Patient demographics are presented in Table 1.

**TABLE 1:** Demographic information of included healthy patients. Values are presented as mean ± SD long‐term cortex culturing (*n* = 6), short‐term cortex culturing (*n* = 3), short‐term medulla culturing (*n* = 2). BMI, body mass index; eGFR, estimated glomerular filtration rate.

Parameter	Medulla (30 min)	Cortex (30 min)	Cortex (48 h)
Gender (% male)	100	66.7	83.3
Age (in years)	52 ± 5	66 ± 14	67 ± 4
BMI	28.5 ± 4.8	28.2 ± 3.7	26.1 ± 3.7
eGFR (mL/min/1.73 m)	90 ± 0	74.7 ± 11.3	80.8 ± 7.8
Ischemia time (min)	20 ± 9	52 ± 49	45 ± 27
Number of patients	2*	3*	6

Abbreviations: BMI, body mass index; eGFR, estimated glomerular filtration rate.

* One patient appears in both groups.

### 2.3 Viability Measurements

The viability of the cortical PCKS used for long‐term evaluation was assessed via measurements of ATP content using the ATP Fluorometric Assay Kit (MAK190, Sigma‐Aldrich) in accordance with the manufacturer's instructions (Figure S1). Results were analyzed using a Kruskal–Wallis test with Dunn's multiple comparisons test.

### 2.4 Multiplex Labeling

#### 2.4.1 Immunohistofluorescence

The multiplex protocol has previously been described [17]. PCKS were sliced in 3 μm thick sections and placed onto coverslips that had been coated in a 0.5% gelatin and 1 mM CrK(SO_4_)_2_*12H_2_O solution to produce a surface that was suitable for stable tissue attachment (protocol is from bio‐techne), similar to superfrost microscope slides. The coverslips were heated to 60°C for 20 min to promote tissue attachment and placed in xylene overnight to dissolve the paraffin. The tissue was then rehydrated in a series of decreasing concentrations of ethanol (3 × 5 min in 99% ethanol, 2 × 5 min in 96% ethanol, and 1 × 5 min in 70% ethanol). The tissue was rinsed 3–4 times in deionized H_2_O, and antigen retrieval was performed by boiling the tissue for 10 min in a microwave oven in TEG buffer (10 mM Tris, 0.5 mM EGTA, pH 9.0). After cooling to RT on ice (approximately 30 min), the tissue was incubated in a shielding buffer (50 mM NH_4_Cl in PBS, pH 7.4) for 30 min to shield aldehyde groups. The tissue was then incubated in blocking buffer (1% BSA, 0.2% gelatin, 0.05% saponin in PBS, pH 7.4) for 3 × 10 min using drop incubation (i.e., placing the coverslip face down onto a drop of buffer on parafilm). The tissue was incubated with the primary antibodies: anti‐AQP2 (newly generated in rabbit against the COOH‐terminal tail domain of rat AQP2, Covalab), anti‐uromodulin (cat# AB733, Merck‐Millipore, 1:500), anti‐Na/K‐ATPase (cat# 05–369, Millipore, AH‐Diagnostics, 1:100), anti‐ZO‐1 (cat# 61–7300, Thermo Scientific, 1:100), anti‐E‐cadherin (cat# sc‐8426, Santa Cruz, 1:200), or anti‐N‐cadherin (cat# ab18203, Abcam, 1:100), diluted in staining buffer (0.1% BSA, 0.3% Triton X‐100 in PBS pH 7.4), first for 30 min at RT and then overnight at 4°C in a humidified chamber. After adjusting to RT for 30 min, the tissue was washed 3 × 5 min in PBS and incubated with the secondary antibodies: anti‐rabbit‐660 (cat# SAB4600193, Sigma, 1:500) or anti‐mouse‐660 (cat# A21055, Invitrogen, 1:500) and 2 μg/mL Hoechst 33,342 (Nuclei counterstain, Thermo Fisher Scientific, Waltham, MA, USA) diluted in staining buffer for 45 min at RT. Finally, the tissue was washed 3 × 5 min in PBS. Samples proceeding to multiplex were kept in PBS at 4°C until imaging, covered with tin foil, and samples not proceeding were mounted on objective glasses using Glycergel (cat# C056330‐2, Agilent Dako).

#### 2.4.2 Stripping of Antibodies

Following imaging, the coverslips used for multiplex were transferred to a new 6‐well plate with TBS‐Ts (1 × TBS with 0.01% Tween‐20 and 100 mM sucrose). The stripping buffer (0.1 M Tris, 2% SDS, 10 mM DTT) was prepared fresh each time in the fume hood, and DTT was thawed and added immediately before use. The tissue was incubated with the stripping buffer at 60°C for 1 h. Finally, the tissue was washed 5 × 5 min in TBS‐Ts and transferred to PBS. Restaining of the tissue was performed starting from the blocking step.

### 2.5 Microscopy

The coverslips were placed in an imaging chamber, originally intended for live imaging (Chamlide magnetic chamber; Live Cell Instrument Co, Republic of Korea), and covered with PBS. The tissue was imaged using either an inverted fluorescence microscope (Nikon Eclipse Ti2) equipped with a 60× or 100× oil objective (2 × 2 binning). Images were analyzed using ImageJ (NIH) [19].

### 2.6 Use of AI for Text Editing

ChatGPT (OpenAI) was used for language editing and refinement of the manuscript text. The tool was used in a closed interaction format. All scientific content, data interpretation, and final editorial decisions were performed and approved by the authors.

## 3 Results

### 3.1 dDAVP‐Induced Aquaporin‐2 Plasma Membrane Targeting in PCKS From Human Renal Medulla

**FIGURE 1:**
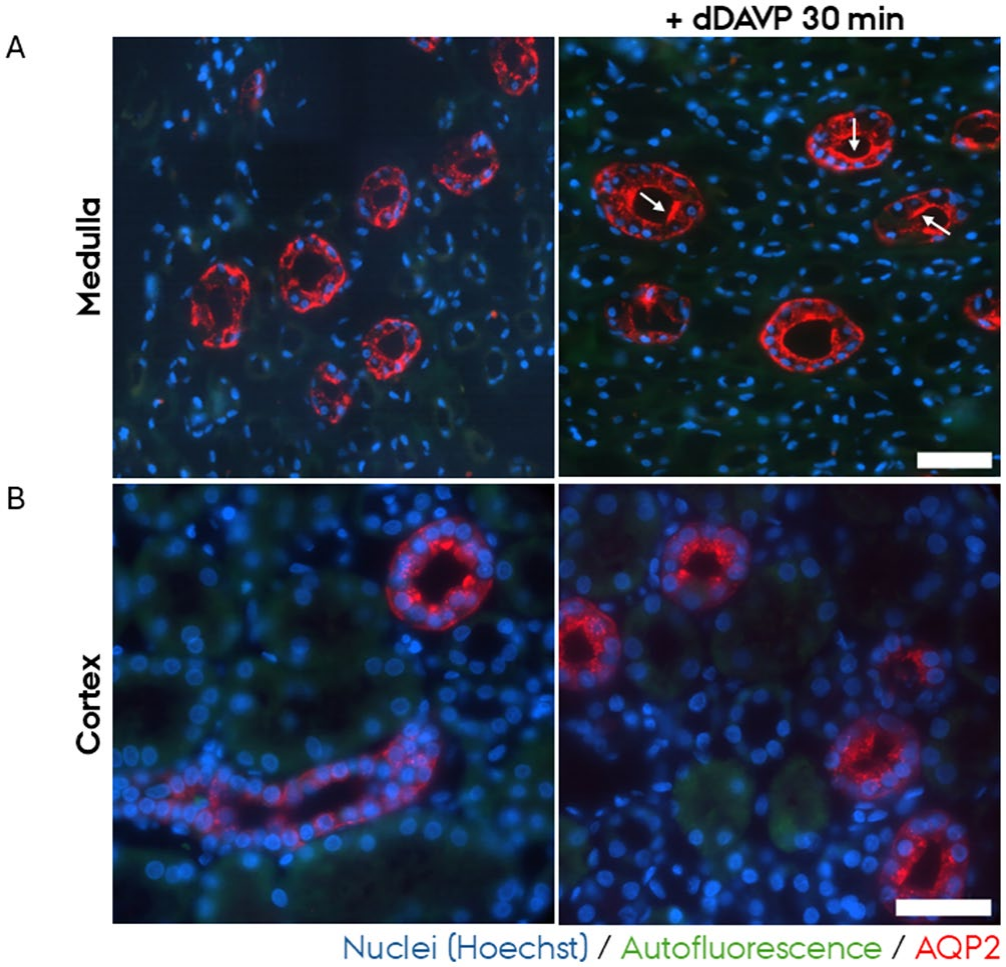
Apical AQP2 plasma membrane accumulation was increased in inner medullary collecting ducts in human precision‐cut kidney slices (PCKS) following 30 min dDAVP treatment. In cortical PCKS, high baseline apical AQP2 localization limited the detection of additional redistribution after dDAVP. Representative images of AQP2 (red) immunofluorescent stainings of medullary (A) and cortical PCKS (B) of untreated PCKS (left) and PCKS treated for 30 min with dDAVP (right). Nuclei were stained with Hoechst (blue). Endogenous tissue autofluorescence (green) was included to delineate tissue morphology and tubular structures. The images were acquired using a Nikon Eclipse Ti‐E system (inverted widefield fluorescence microscope) with a 100× oil objective and binning 2 × 2. Brightness and contrast were adjusted linearly. Arrows in (A) indicate AQP2 accumulation in the apical plasma membrane following dDAVP treatment. Scale bars: 50 μm.

To evaluate if human PCKS could be used to study rapid cellular signaling, PCKS from the medullary and cortical regions were incubated for 30 min with or without dDAVP. dDAVP induces the signaling cascade that results in apical accumulation of AQP2 by facilitating exocytosis of AQP2‐bearing intracellular vesicles [7] and decreasing apical AQP2 endocytosis [20]. In medullary PCKS treated with the vehicle, immunohistofluorescence revealed that AQP2 localized intracellularly and to the basolateral plasma membrane with no prominent targeting to the apical plasma membrane. Upon 30 min of dDAVP treatment, there was a pronounced accumulation of AQP2 at the apical plasma membrane, indicating a rapid cellular response to dDAVP (Figure 1A). In cortical PCKS, pronounced apical AQP2 accumulation, as well as basolateral and cytoplasmic localization, was observed in the unstimulated PCKS, hindering evaluation of the effect of dDAVP on apical AQP2 plasma membrane accumulation (Figure 1B). Together, these data show that medullary PCKS retain a rapid dDAVP‐responsive AQP2 redistribution, consistent with preserved short‐term signaling. In cortical PCKS, the high baseline apical AQP2 signal precluded assessment of further redistribution.

### 3.2 Incubation of PCKS for 24 h and 48 h Results in Mislocalization of AQP2, the Na/K‐ATPase and Uromodulin

**FIGURE 2:**
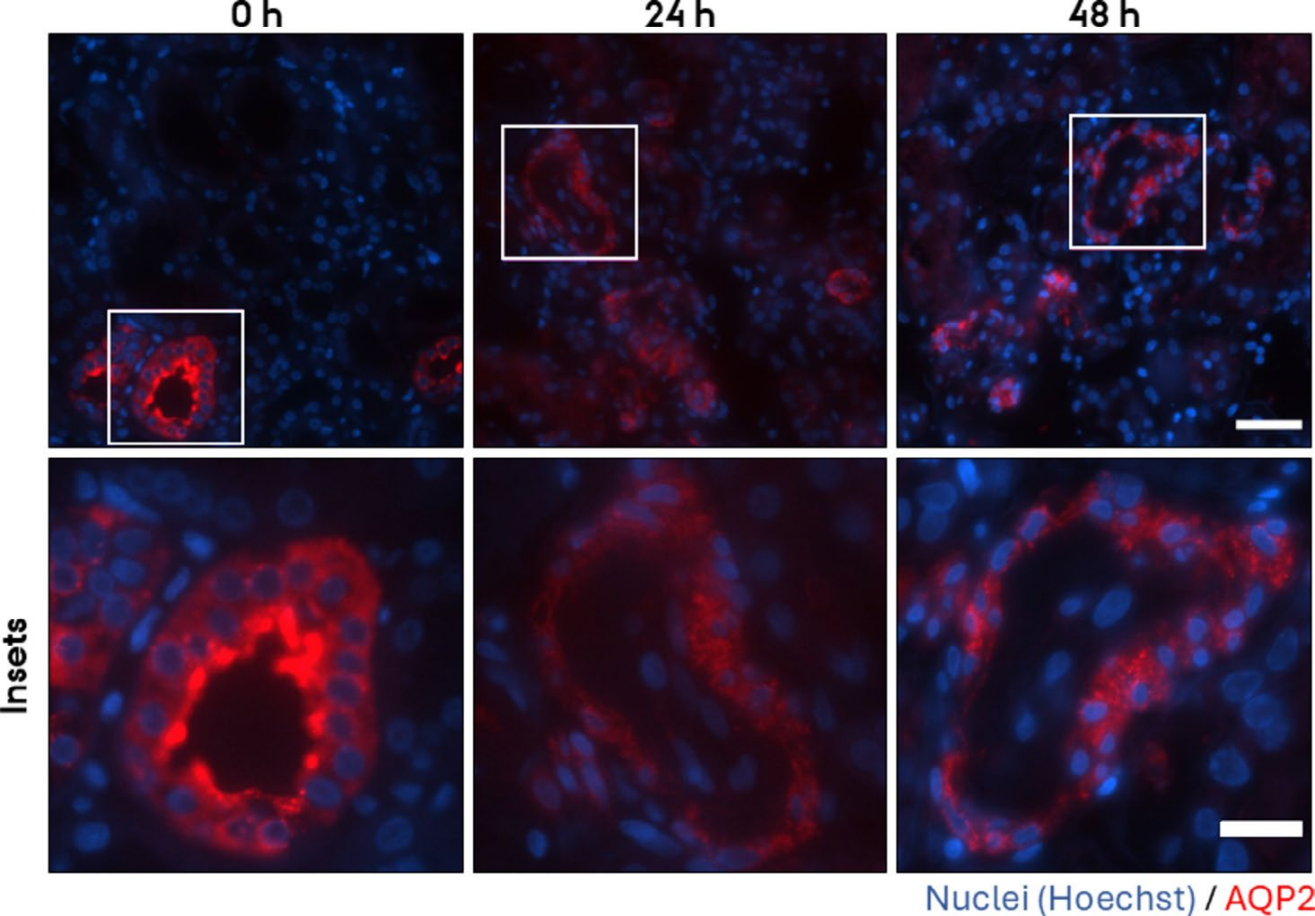
AQP2 subcellular localization was dysregulated in cortical human precision‐cut kidney slices (PCKS) following 24 and 48 h incubation, showing a progressive loss of apical membrane labeling and increased intracellular signal. Representative images of immunofluorescent stainings of AQP2 (red) of PCKS incubated for 0 h, 24 h, and 48 h. Nuclei were stained with Hoechst (blue). The images were acquired using a Nikon Eclipse Ti‐E system (inverted widefield fluorescence microscope) with a 100× oil objective and binning 2 × 2. Brightness and contrast were adjusted linearly. Insets (lower panel) show higher magnification of the boxed areas. Scale bars: 50 μm (upper panel) and 20 μm (insets).

**FIGURE 3:**
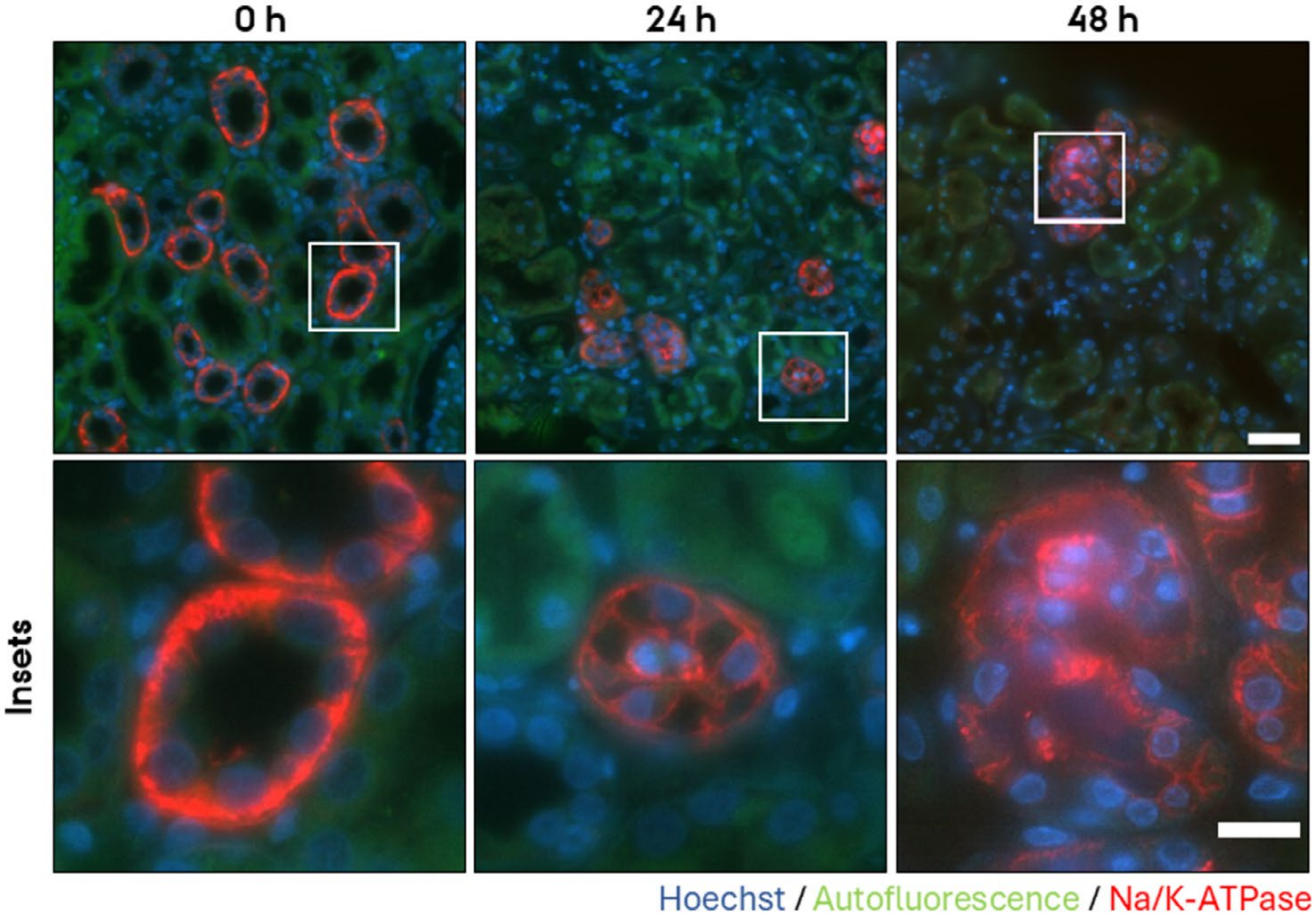
Na/K‐ATPase subcellular localization was dysregulated in cortical human precision‐cut kidney slices (PCKS) following 24 and 48 h incubation, showing progressive loss of basolateral labeling and increased apical signal. Representative images of immunofluorescent stainings of the Na/K‐ATPase (red) of PCKS incubated for 0 h, 24 h, and 48 h. Nuclei were stained with Hoechst (blue). Endogenous tissue autofluorescence (green) was included to delineate tissue morphology and tubular structures. The images were acquired using a Nikon Eclipse Ti‐E system (inverted widefield fluorescence microscope) with a 60× oil objective and binning 2 × 2. Brightness and contrast were adjusted linearly. Insets (lower panel) show higher magnification of the boxed areas. Scale bars: 50 μm (upper panel) and 20 μm (insets).

**FIGURE 4:**
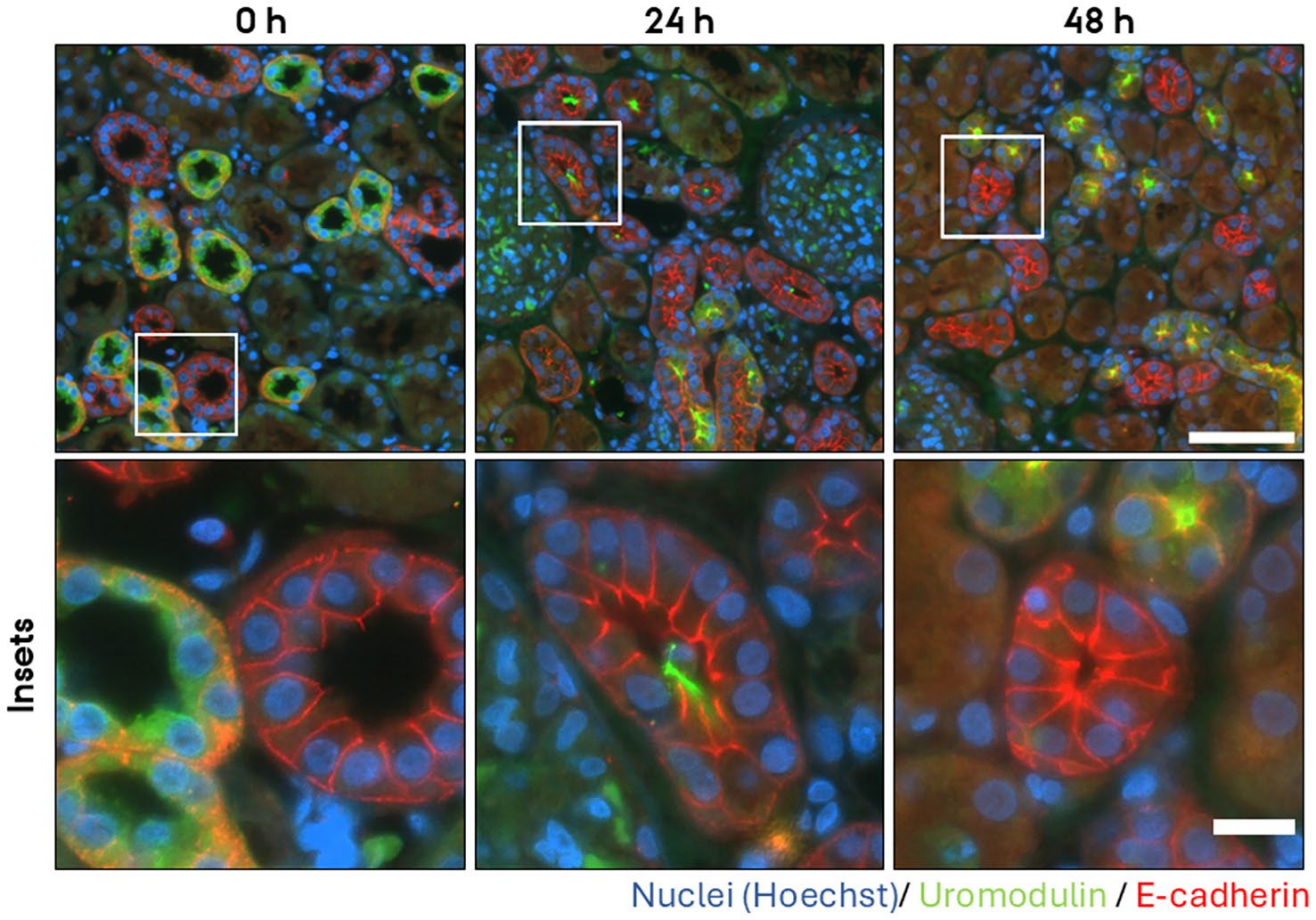
Uromodulin and E‐cadherin subcellular localizations were dysregulated in cortical human precision‐cut kidney slices (PCKS) following 24 and 48 h incubation. Uromodulin showed progressive loss of intracellular labeling and increased luminal localization, whereas E‐cadherin showed progressive loss of basolateral labeling and increased apical signal. Representative images of immunofluorescent stainings of Uromodulin (green) and E‐cadherin (red) of PCKS incubated for 0 h, 24 h, and 48 h. Nuclei were stained with Hoechst (blue). The images were acquired using a Nikon Eclipse Ti‐E system (inverted widefield fluorescence microscope) with a 60× oil objective and binning 2 × 2. Brightness and contrast were adjusted linearly. Insets (lower panel) show higher magnification of the boxed areas. Scale bars: 50 μm (upper panel) and 20 μm (insets).

To investigate if the PCKS maintain epithelial function after prolonged incubation, we investigated the subcellular localization of three prominent renal transport proteins in the cortex, namely AQP2, which is expressed in collecting ducts, Na/K‐ATPase, which is especially strongly expressed in proximal tubules and thick ascending limbs of Henle (TAL), and uromodulin expressed in TAL. Before culture, AQP2 localized to both the apical and basolateral plasma membrane and in the cytoplasm; however, after culturing for 24 h and 48 h, AQP2 localized mainly intracellularly (Figure 2). Before incubation, Na/K‐ATPase localized to the basolateral plasma membrane; however, after culturing for 24 h and 48 h, the localization was uniform in both the apical and basolateral plasma membranes (Figure 3). Also, uromodulin was mislocalized from an intracellular localization (Figure 4, green channel) to a luminal localization. Thus, these mislocalizations suggest that epithelial function is disrupted upon 24 h and 48 h of culturing.

### 3.3 Incubation of the PCKS Resulted in Alteration of Epithelial Junctions

**FIGURE 5:**
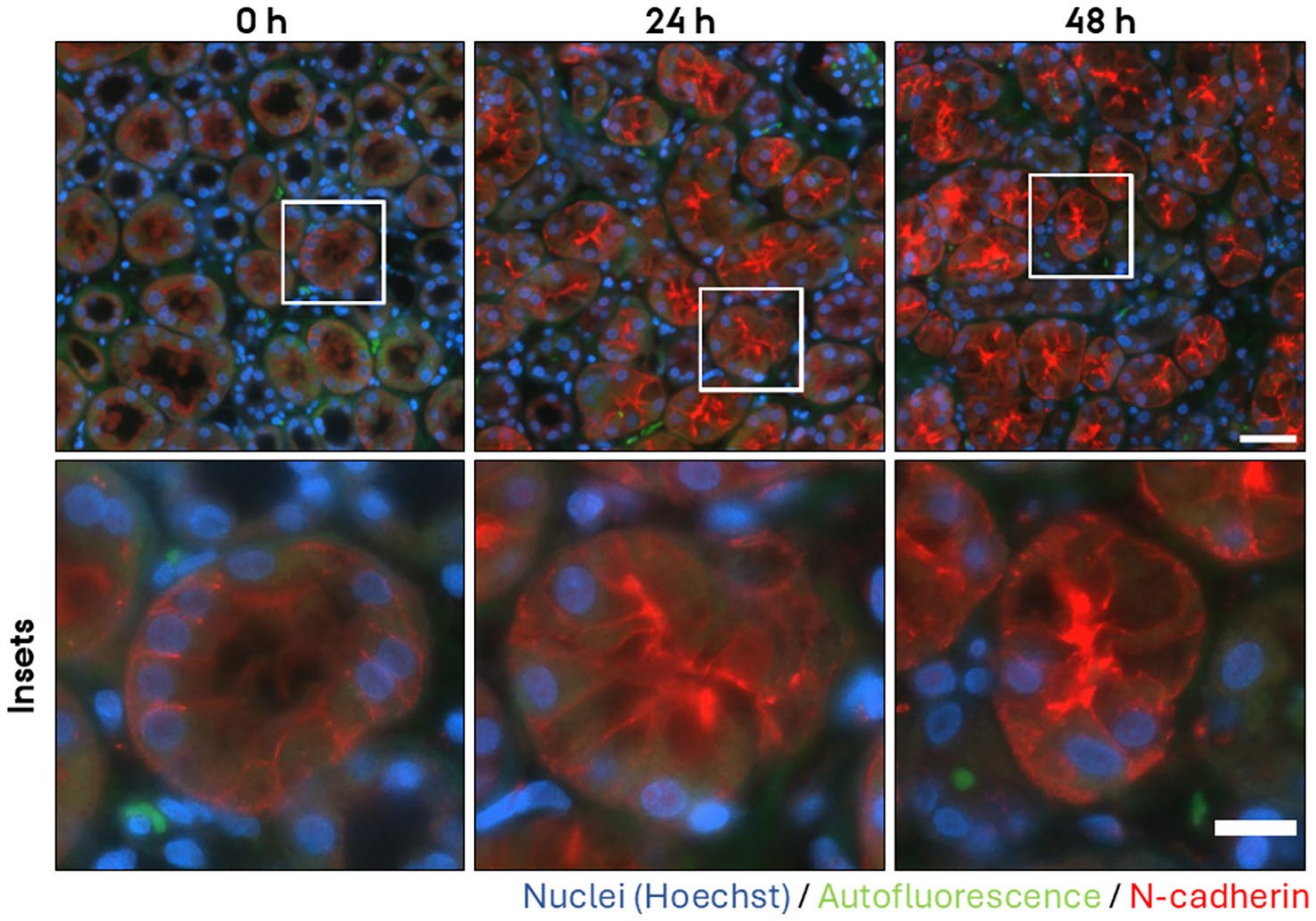
N‐cadherin subcellular localization was dysregulated in cortical human precision‐cut kidney slices (PCKS) following 24 and 48 h incubation, showing progressive loss of basolateral labeling and increased apical signal. Representative images of immunofluorescent stainings of N‐cadherin (red) of PCKS incubated for 0 h, 24 h, and 48 h. Nuclei were stained with Hoechst (blue). Endogenous tissue autofluorescence (green) was included to delineate tissue morphology and tubular structures. The images were acquired using a Nikon Eclipse Ti‐E system (inverted widefield fluorescence microscope) with a 60× oil objective and binning 2 × 2. Brightness and contrast were adjusted linearly. Insets (lower panel) show higher magnification of the boxed areas. Scale bars: 50 μm (upper panel) and 20 μm (insets).

**FIGURE 6:**
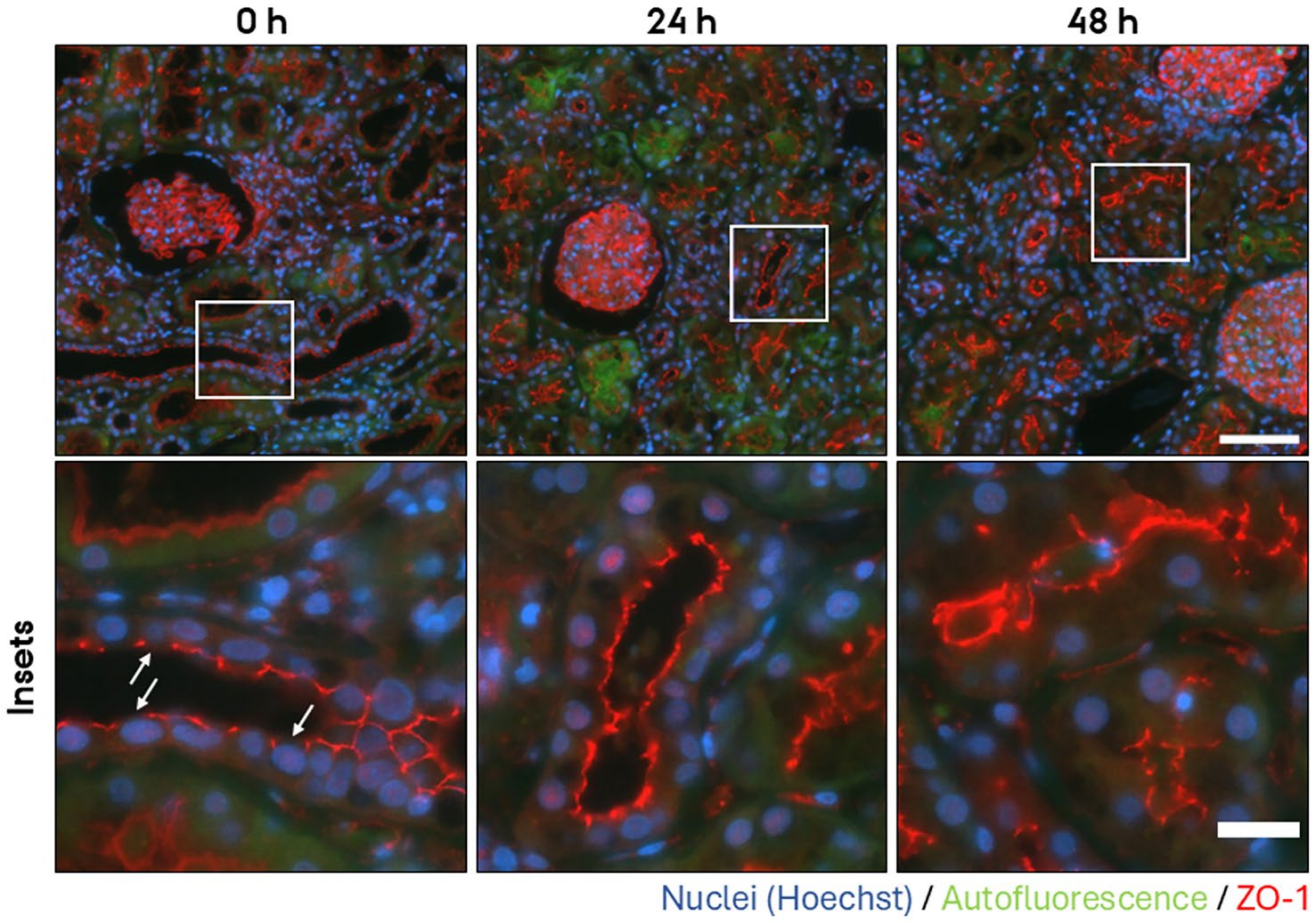
ZO‐1 subcellular localization was dysregulated in cortical human precision‐cut kidney slices (PCKS) following 24 and 48 h incubation, showing a progressive loss of labeling at the apical cell–cell junctions and redistribution to the apical membrane. Representative images of immunofluorescent stainings of ZO‐1 (red) of PCKS incubated for 0 h, 24 h, and 48 h. Nuclei were stained with Hoechst (blue). Endogenous tissue autofluorescence (green) was included to delineate tissue morphology and tubular structures. The images were acquired using a Nikon Eclipse Ti‐E system (inverted widefield fluorescence microscope) with a 60× oil objective and binning 2 × 2. Brightness and contrast were adjusted linearly. Insets (lower panel) show higher magnification of the boxed areas. Arrows point to the apical plasma membrane without ZO‐1 localization. Scale bars: 50 μm (upper panel) and 20 μm (insets).

**FIGURE 7:**
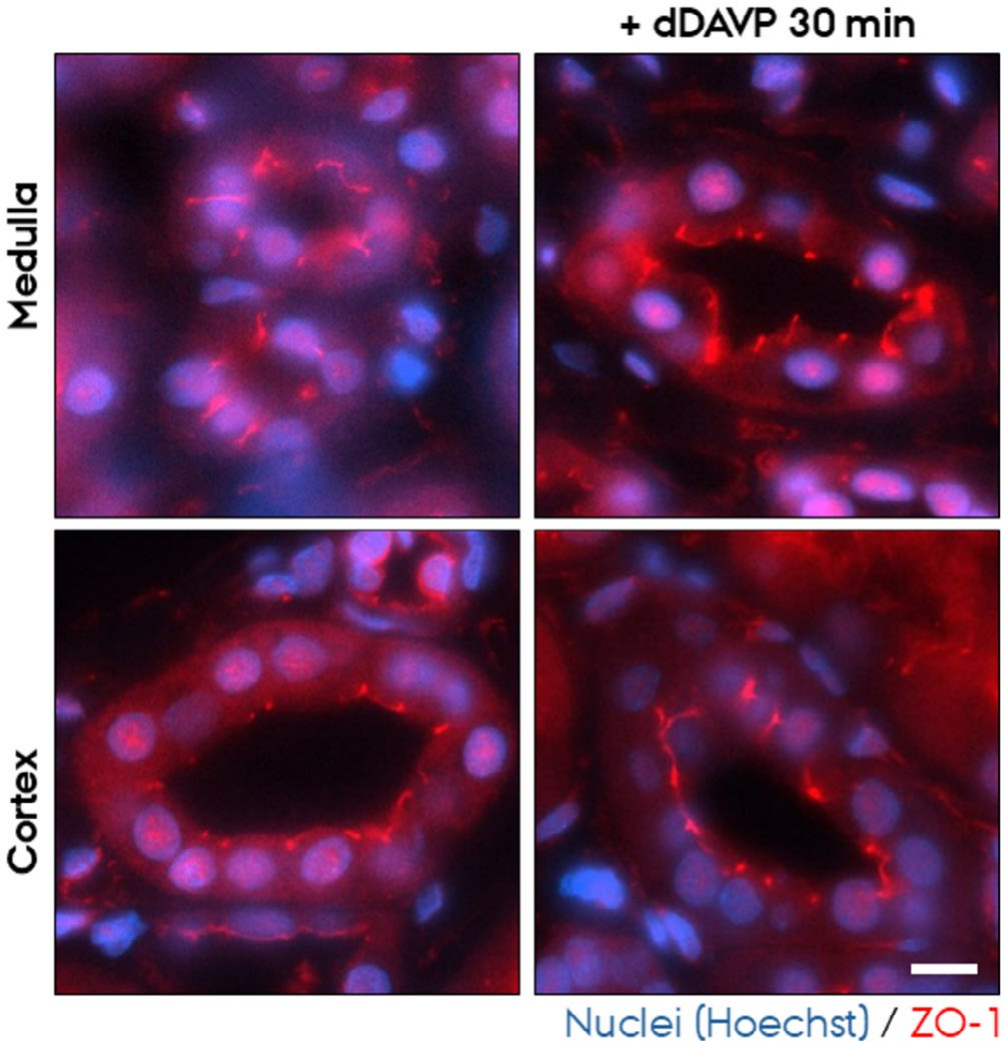
ZO‐1 subcellular localization was unaltered in cortical and medullary human precision‐cut kidney slices (PCKS) following 30 min incubation with or without dDAVP. Representative images of immunofluorescent staining of ZO‐1 (red) of PCKS incubated for 30 min with or without dDAVP. Nuclei were stained with Hoechst (blue). The images were acquired using a Nikon Eclipse Ti‐E system (inverted widefield fluorescence microscope) with a 60× oil objective and binning 2 × 2. Brightness and contrast were adjusted linearly. Scale bar: 10 μm.

To assess whether the PCKS maintain epithelial architecture, we examined the subcellular localization of key components of tight‐ and adherens junctions in the cortical region after 24 h and 48 h culturing. For adherens junctions, we investigated the localization of E‐cadherin and N‐cadherin. N‐cadherin is expressed in the proximal tubules, and E‐cadherin in the remaining segments of the nephron and collecting ducts. In freshly cut slices (0 h), E‐cadherin and N‐cadherin localized laterally to cell–cell contacts as well as to the basolateral plasma membrane (Figure 4, red channel [E‐cadherin] and Figure 5 [N‐cadherin]). Upon culturing for 24 h and 48 h, both E‐cadherin and N‐cadherin mislocalized. E‐cadherin localized to all plasma membrane domains (Figure 4, red channel), and apical localization of N‐cadherin became prominent (Figure 5). As a marker for tight junctions, we investigated the localization of zonula occludens 1 (ZO‐1). At 0 h, ZO‐1 localized sharply to the apex of epithelial cells at the position of tight junctions (Figure 6), whereas the apical membrane was negative for ZO‐1 staining. By 24 h and 48 h, this pattern was lost, and ZO‐1 became progressively redistributed across the entire apical membrane (Figure 6). Of note, 30 min incubation did not noticeable alter ZO‐1 localization (Figure 7).

## 4 Discussion

This study demonstrates that human medullary PCKS retain key aspects of short‐term epithelial signaling, as evidenced by the rapid trafficking of aquaporin‐2 (AQP2) to the apical plasma membrane upon desmopressin (dDAVP) stimulation in medullary PCKS. Due to the high AQP2 apical accumulation in cortical slices, the effect of dDAVP could not be assessed, but the localization of the tight junction marker ZO‐1 was unaltered by 30 min incubation in both medullary and cortical PCKS. In contrast to the short‐term incubated PCKS, prolonged culturing (24 h and 48 h) of cortical PCKS led to mislocalization of key transport proteins and epithelial junctional proteins, indicating loss of epithelial architecture over time.

The response of AQP2 to dDAVP stimulation in medullary PCKS confirms that medullary PCKS are well‐suited to study rapid signaling events, such as hormone‐regulated AQP2 exocytosis. Previous studies have relied on immortalized cell lines or rodent models [9, 10, 11, 12, 13, 14, 15], including isolated collecting ducts [7] and rodent kidney slices [16], to investigate AQP2 shuttling, but these models have limitations in faithfully replicating human physiology. The PCKS system, by preserving human cellular diversity and native tissue architecture, provides a complementary human ex vivo model. This makes PCKS a valuable tool for studying human‐specific aspects of short‐term signaling, particularly in the context of pharmacological interventions targeting protein trafficking or other short‐term mechanisms. However, access to medullary tissue in human nephrectomy samples is limited, which constrains the availability of medullary PCKS for experimental studies. Therefore, assessment of epithelial integrity after 24 and 48 h incubation was performed for cortical PCKS.

While PCKS maintain epithelial signaling within a 30 min window following ischemia and handling time, our findings highlight limitations in their ability to sustain epithelial integrity at 24 h and 48 h culturing, although tissue viability is preserved. The mislocalization of AQP2, NaK‐ATPase, and uromodulin suggests an alteration of epithelial transport processes. Similarly, altered localization of ZO‐1, E‐cadherin, and N‐cadherin indicates compromised tight and adherens epithelial junctional organization and thus, cellular polarity. The absence of physiological factors such as blood flow, mechanical forces, and osmotic gradients likely contributes to the observed epithelial alterations during prolonged culturing. Despite their limitations for long‐term epithelial function studies, PCKS remain a powerful tool for investigating disease mechanisms, particularly in the context of renal fibrosis and inflammation, as previous studies have successfully induced fibrotic and inflammatory responses in PCKS using transforming growth factor‐beta (TGF‐β) [2, 3] and tumor necrosis factor‐alpha (TNF‐α), respectively [6]. The ability to manipulate these processes in a human‐derived system allows for mechanistic studies of renal pathology and preclinical drug testing. Yet, a limitation of the model is the relatively short viability of the slices. This could potentially be improved by optimizing culture conditions, particularly the composition of the culture medium. Some progress has already been made in this direction [21, 22, 23]. Extending the longevity of the slices would further increase the applicability of the model for studying a range of (patho)physiological processes.

Given that nearly 90% of drugs entering clinical trials fail to reach approval [1], there is a critical need for more predictive human‐based model systems. PCKS provide an opportunity to assess drug effects on human renal tissue in a controlled setting, bridging the gap between in vitro cell‐based assays and in vivo animal studies. The short‐term preservation of cellular signaling in PCKS further supports their use in evaluating acute drug responses that, e.g., target signal‐mediated regulated exocytosis.

In conclusion, the model of human PCKS offers a relevant ex vivo model for studying cellular signaling and for testing different pharmacological interventions. However, the limitations in sustaining long‐term epithelial architecture must be considered.

## Funding

This work was supported by an ODIN (the Open Discovery Innovation Network) grant supported by the Novo Nordisk Foundation (Grant No. NNF20SA0061466) towards the FRIGG project (FResh Human KIdney Tissue: ExplorinG biomarkers and intervention tarGets in chronic kidney disease).

## Conflicts of Interest

The authors declare no conflicts of interest.

## Supporting information


**Figure S1:** Viability of the cortical PCKS at 0 h, 24 h, and 48 h assessed by ATP measurements (*n* = 6). No significant difference in ATP levels was observed between the three timepoints (Kruskal–Wallis test with Dunn's multiple comparisons test).

## Data Availability

The data that support the findings of this study are available on request from the corresponding author. The data are not publicly available due to privacy or ethical restrictions.
