# Supplementary figures and images for: Evaluation of Epithelial Integrity in Human Precision‐Cut Kidney Slices
Source: APMIS. 2026 Apr 10;134(4):e70205. doi: 10.1111/apm.70205 (PMC13068632; doi:10.1111/apm.70205)

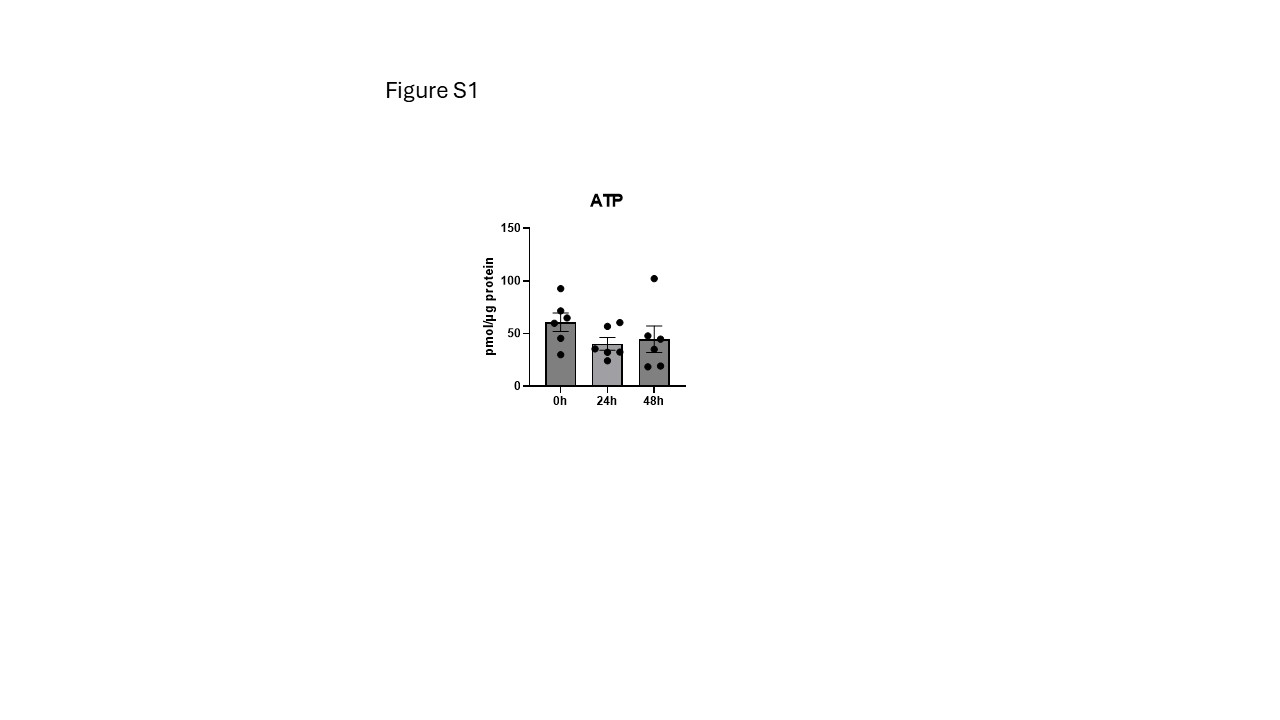

Supplement: Supplementary file 1 — Figure S1: Viability of the cortical PCKS at 0 h, 24 h, and 48 h assessed by ATP measurements (n = 6). No significant difference in ATP levels was observed between the three timepoints (Kruskal–Wallis test with Dunn's multiple comparisons test). [file APM-134-0-s001.jpg]
